# Prevention and Control of Influenza with Vaccines: Interim Recommendations of the Advisory Committee on Immunization Practices (ACIP), 2013

**Published:** 2013-05-10

**Authors:** Wendy Keitel, Lisa Grohskopf, Joseph Bresee, Nancy Cox, Leslie Sokolow

**Affiliations:** Baylor College of Medicine, Houston, TX; Influenza Div, National Center for Immunization and Respiratory Diseases, CDC

This report summarizes recommendations approved on February 21, 2013, by the Advisory Committee on Immunization Practices (ACIP) for the use of influenza vaccines. An expanded 2013 ACIP influenza vaccination recommendation statement is scheduled to be published in *MMWR Recommendations and Reports* before the start of the 2013–14 influenza season. Providers should consult the expanded 2013 ACIP influenza vaccination statement for complete and updated information.

## Vaccine Recommendations

Routine annual influenza vaccination is recommended for all persons aged ≥6 months. Immunization providers should consult Food and Drug Administration–approved prescribing information for 2013–14 influenza vaccines and the 2013–14 ACIP influenza recommendation statement for the most current information concerning indications, contraindications, and precautions.

Recommendations for routine use of vaccines in children, adolescents and adults are developed by the Advisory Committee on Immunization Practices (ACIP). ACIP is chartered as a federal advisory committee to provide expert external advice and guidance to the Director of the Centers for Disease Control and Prevention (CDC) on use of vaccines and related agents for the control of vaccine-preventable diseases in the civilian population of the United States. Recommendations for routine use of vaccines in children and adolescents are harmonized to the greatest extent possible with recommendations made by the American Academy of Pediatrics (AAP), the American Academy of Family Physicians (AAFP), and the American College of Obstetrics and Gynecology (ACOG). Recommendations for routine use of vaccines in adults are harmonized with recommendations of AAFP, ACOG, and the American College of Physicians (ACP). ACIP recommendations adopted by the CDC Director become agency guidelines on the date published in the *Morbidity and Mortality Weekly Report* (*MMWR*). Additional information regarding ACIP is available at http://www.cdc.gov/vaccines/acip.

## Available Influenza Vaccines for 2013–14

Influenza vaccines that are currently licensed and expected to be available for the 2013–14 season and their approved age indications are summarized in a table available at http://www.cdc.gov/flu/professionals/acip/2013-interim-recommendations.htm#table1. The information in the table is current as of April 15, 2013. Any changes in product availability or other information will be reflected in the expanded 2013–14 ACIP influenza recommendations statement. The table lists four newly licensed influenza vaccines that are expected to be available during the 2013–14 influenza season. These vaccines are acceptable alternatives to other licensed products listed in the table, to the extent that their specific indications allow. For persons for whom more than one type of vaccine is appropriate and available, ACIP does not express a preference for use of any particular product over another.

## Note on Influenza Vaccine Abbreviations

Certain U.S. vaccine abbreviations have been revised by ACIP to refer to currently available influenza vaccines.* The revisions are as follows:

- The abbreviation TIV (trivalent influenza vaccine, previously used for inactivated influenza vaccines) has been replaced with the abbreviation IIV (inactivated influenza vaccine). For 2013–14, IIVs as a class will include 1) egg-based and cell culture-based trivalent inactivated influenza vaccine (IIV3), and 2) egg-based quadrivalent inactivated influenza vaccine (IIV4).
- RIV refers to recombinant hemagglutinin influenza vaccine, which will be available as a trivalent formulation (RIV3) for 2013–14.
- LAIV refers to live, attenuated influenza vaccine, which will be available as a quadrivalent formulation (LAIV4) for 2013–14.
- LAIV, IIV, and RIV denote vaccine categories; a numeric suffix specifies the number of influenza virus antigens contained in the vaccine.
- Where necessary to refer specifically to cell culture-based vaccine, the prefix “cc” is used (e.g., “ccIIV3”).

## Footnotes

* Additional information available at http://www.cdc.gov/vaccines/acip/committee/guidance/vac-abbrev.html.

